# Emerging functions of circular RNA in the regulation of adipocyte metabolism and obesity

**DOI:** 10.1038/s41420-022-01062-w

**Published:** 2022-05-20

**Authors:** Yuanyuan Zhang, Zhichen Tian, Haibo Ye, Xiaomei Sun, Huiming Zhang, Yujia Sun, Yongjiang Mao, Zhangping Yang, Mingxun Li

**Affiliations:** 1 Joint International Research Laboratory of Agriculture and Agri-Product Safety, the Ministry of Education of China, Yangzhou University, 225009 Yangzhou, Jiangsu China; 2 Key Laboratory of Animal Genetics & Breeding and Molecular Design of Jiangsu province, College of Animal Science and Technology, Yangzhou University, 225009 Yangzhou, Jiangsu China

**Keywords:** Long non-coding RNAs, Gene regulation

## Abstract

As noncoding RNAs, circular RNAs (circRNAs) are covalently enclosed endogenous biomolecules in eukaryotes that have tissue specificity and cell specificity. circRNAs were once considered a rare splicing byproduct. With the development of high-throughput sequencing, it has been confirmed that they are expressed in thousands of mammalian genes. To date, only a few circRNA functions and regulatory mechanisms have been verified. Adipose is the main tissue for body energy storage and energy supply. Adipocyte metabolism is a physiological process involving a series of genes and affects biological activities in the body, such as energy metabolism, immunity, and signal transmission. When adipocyte formation is dysregulated, it will cause a series of diseases, such as atherosclerosis, obesity, fatty liver, and diabetes. In recent years, many noncoding RNAs involved in adipocyte metabolism have been revealed. This review provides a comprehensive overview of the basic structure and biosynthetic mechanism of circRNAs, and further discusses the circRNAs related to adipocyte formation in adipose tissue and liver. Our review will provide a reference for further elucidating the genetic regulation mechanism of circRNAs involved in adipocyte metabolism.

## Facts

- Adipocyte metabolism is a complex physiological process involving a series of genes and regulatory factors.
- circRNAs are involved in adipogenesis and lipolysis in the adipose tissue and the liver.
- circRNAs can act as miRNA sponges, transcriptional regulators, protein scaffolds, or translation templates.

## Open questions

- Whether or not circRNAs have a biological function in adipocyte metabolism?
- How do genes, miRNAs, and circRNAs coordinately regulate adipocyte metabolism?
- Can circRNAs be used as biomarkers and as therapeutic carriers?

## Introduction

Adipocyte metabolism is a complex physiological process involving nutrition regulation, hormone regulation, and homeostasis. It participates in a variety of physiological processes and is of great significance to the life activities of the body. Abnormal adipocyte metabolism and metabolic disorders have become epidemics in various countries around the world [1, 2], and can cause metabolic syndromes, including obesity, hepatic steatosis, adipose tissue dysfunction, atherosclerosis, and type 2 diabetes [3, 4]. Studies have shown that peroxisome proliferator-activated receptor γ (PPARγ) [5], CCAAT/enhancer-binding protein (C/EBPα) [6], and the sterol regulatory element-binding protein (SREBP) family [7] participate in the adipocyte metabolism regulation. However, an increasing number of noncoding RNAs (ncRNAs), including microRNAs (miRNAs) [8–10], long noncoding RNAs (lncRNAs) [11–14], and circular RNAs (circRNAs) [15, 16], have been shown to be involved in the regulation of adipocyte metabolism [17]. These ncRNAs regulate adipogenesis in a variety of ways [18].

ncRNAs are a general term for functional RNA that does not code for proteins. They participate in gene expression regulation through epigenetic modification, transcriptional, and posttranscriptional regulation [19]. Among them, circRNAs are an emerging type of ncRNA with great research potential, following miRNAs and lncRNAs in the noncoding RNA family. circRNAs are a class of covalently enclosed endogenous biomolecules that were first discovered in 1976 [20]. circRNAs play important roles in the growth or development of tissues and organs, as they are involved in cell cycle regulation, cell proliferation, and apoptosis in different tissues or organs [21–25]. In the past few years, the biosynthesis and molecular functions of circRNAs have been extensively explored and verified. circRNAs can act as miRNA sponges to adsorb and regulate the activity of miRNAs, and can also modulate the transcription and translation of downstream target genes by binding to proteins [26].

The expression levels of circRNAs in tissues are low, with tissue-specific and cell-specific expression patterns [27–29]. Although circRNAs are closely related to a series of physiological processes in animals, the biogenesis mechanism of circRNAs in different animals has certain differences [30]. Previous studies have confirmed that circRNAs are involved in adipogenesis and lipolysis in adipose tissue, and also play regulatory roles in lipid metabolism in hepatocytes [31]. Dysregulation of circRNAs is closely related to the occurrence of a series of diseases, such as adipogenesis-related disorders, diabetes, and metabolic diseases [32, 33]. Therefore, in this paper, we have reviewed the basic structure and biosynthesis mechanism of circRNAs, and further discuss the regulatory roles of circRNAs in adipocyte metabolism. Our review will provide a reference for further elucidating the genetic regulation mechanism of circRNAs involved in adipocyte metabolism.

## Properties and biogenesis of circRNAs

### Structure of circRNAs

**Fig. 1:**
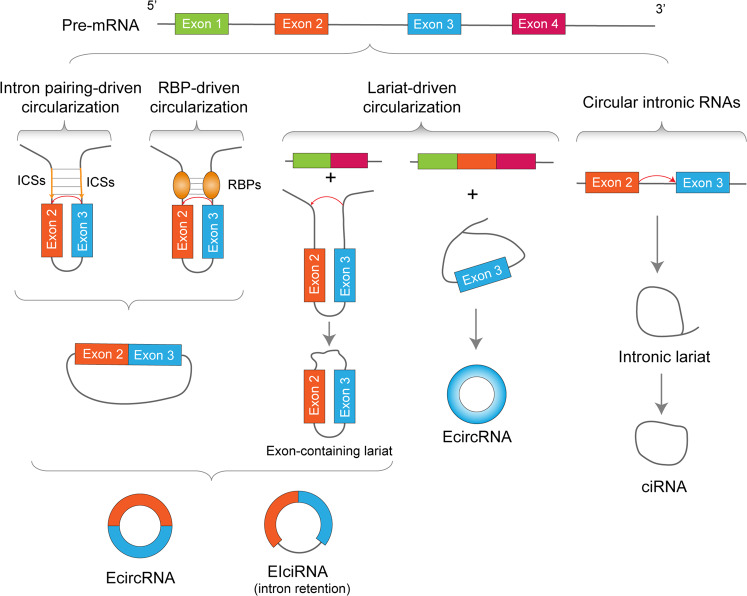
The biogenesis of circRNAs. Base pairing between intronic reverse complementary sequences (ICSs) of the flanking introns or the RBPs dimerization prompts the 3′ splice donor site joined with the 5′ splice acceptor site, leading to the backsplicing. This event results in the formation of exon–intron circRNAs (EIcircRNAs) or exonic circRNAs (EcircRNAs). Alternatively, circRNAs can also arise from exon-containing lariats or intronic lariats.

circRNAs do not have a 5′-end cap or a 3′-end poly(A) tail and forms a closed-loop structure with covalent bonds. Therefore, circRNAs are not easily affected by RNA exonuclease, their expressions are more stable, and are not easily degraded [34, 35]. With the rapid development of high-throughput sequencing and bioinformatics analysis, thousands of circRNAs have been identified in the cells and tissues of different species. It has been discovered that circRNAs can be produced from intergenic regions, intronic regions, coding regions, and 5′ or 3′ noncoding regions [36]. According to their location generated in the genome, circRNAs are classified into the following three categories [37, 38]: (1) exonic circRNAs (EcirRNAs), (2) exon–intron circRNAs (EIcircRNAs), and (3) circular intronic RNAs (ciRNAs) (Fig. 1). EcirRNAs are existed in the cytoplasm. They have a similar miRNAs sponge effect to lncRNAs, and can indirectly regulate gene expression by competitively binding to miRNAs [39]. However, EIciRNAs and ciRNAs are abundant in the nucleus, with low enrichment for miRNA targets. EIciRNAs are able to interact with U1 small nuclear ribonucleoprotein (U1 snRNP) to form an EIciRNA–U1 snRNP complex that binds to the RNA polymerase II (Pol II) transcription complex to facilitate the transcription of their parental genes [40]. Inhibition of ciRNAs can result in the decreased expression of their parental genes, although the mechanism of this is not clear [41].

### Biogenesis and characteristics of circRNAs

The biosynthesis mechanisms of different types of circRNAs are different. To date, three different cyclization models have been proposed [38, 42, 43]: intron pairing-driven circularization, RNA-binding protein (RBP)-driven circularization, and lasso-driven circularization (Fig. 1).

The intron pairing-driven circularization is an important mechanism of circRNA biosynthesis. Most circRNAs are generated by back splicing, in which the downstream 5′ splice donor is connected to the upstream 3′ splice acceptor [44]. Some introns on both sides of the circRNA exons contain intronic reverse complementary sequences (ICSs) [45], and these sequences are paired by base pairing to form a circular structure. The side-by-side orientation at the splicing site forms RNA double strands, which is then cut to form two different kinds of circRNAs with or without introns (Fig. 1) [27]. The competitive pairing of complementary pairing sequences of different *cis*-introns can produce multiple circRNAs from one gene locus [41].

The biogenesis of circRNAs can also be driven by RNA-binding proteins (RBPs) (Fig. 1). For example, the known splicing factor Muscleblind can promote the circularization of its second exon by binding to the flanking introns [46]. Quaking is another RBP known to play a role in mRNA splicing, which leads to the formation of circRNA through the combination of recognition elements in its upstream and downstream introns [47].

Lasso-driven circularization is another major mechanism for generating circRNAs. When the pre-mRNA undergoes canonical GU/AG splicing, exon skipping splicing patterns can sometimes occur, resulting in a lasso intermediate containing intron–exon, and then the lasso intermediate is spliced to form circRNAs. ciRNAs are originated from lariat introns. Their genesis relies on a 7 nt GU-rich motif at the 5′ splice site and an 11 nt C-rich motif at the branchpoint site. The intron-containing lasso is cyclized through covalent 2′, 5′-phosphodiester bond, and then the redundant sequence from the 3′ end of the intron to the branch site is degraded (Fig. 1) [41].

## The regulatory mechanism of circRNAs

### Acting as miRNA sponges

**Fig. 2:**
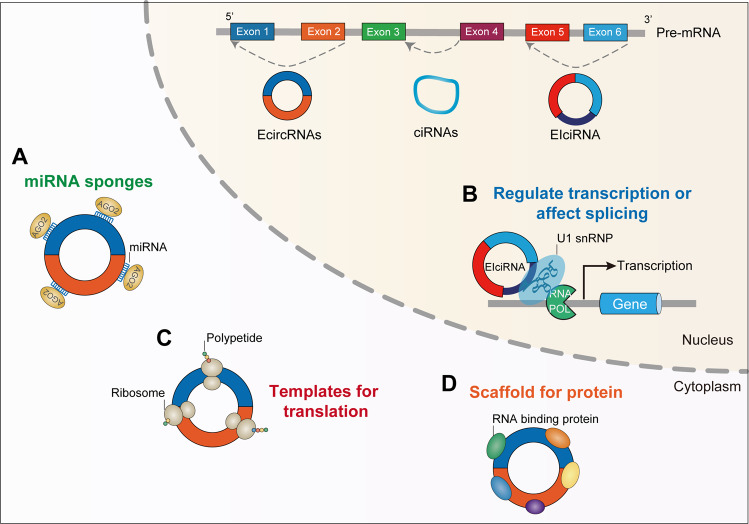
Regulatory mechanisms of circRNAs functions. **A** circRNAs can act as miRNA sponges, decreasing the ability of miRNAs to target mRNAs. **B** circRNAs may recruit the RNA polymerase II (Pol II) and U1 small nuclear ribonucleoprotein (U1 snRNP) to the promoter of the host gene to facilitate their host gene transcription. **C** circRNAs might bound by ribosomes and translate into small peptides. **D** circRNAs can function as RNA-binding proteins (RBPs) scaffolds and indirectly modulate these proteins functions.

Recent studies have shown that circRNA molecules are rich in miRNA-binding sites and can act as miRNA sponges in cells (Fig. 2A). circRNA binds to miRNAs to block the inhibitory effect of the miRNA on its target genes, thereby upregulating the target genes expression. This mechanism of action is called the competitive endogenous RNA (ceRNA) [48]. CiRS-7 was discovered to be a sponge of miR-138, revealing for the first time that circRNAs can act as miRNA sponges [49]. CiRS-7 can also regulate insulin transcription in pancreatic islet cells by adsorbing miR-7, which promotes insulin secretion and increases the insulin levels in pancreatic islet cells [50]. In liver, circRNA-0067835 acts as a sponge of miR-155 to promote the expression of FOXO3a to regulate liver fibrosis [51].

circRNAs not only can interact with a single miRNA but also can regulate the activity of multiple miRNAs at the same time [52]. Hsiao KY et al. [53]. have shown that circCCDC66 plays an oncogenic function in polyps and colon cancer. circCCDC66 contains several binding sites for miR-33b and miR-93. These two miRNAs can target MYC oncogenes and thus accelerate tumor growth and cancer invasion. Because circRNAs are stably expressed in cells and have a large number of miRNA-binding sites, they may be more effective miRNA sponges than linear RNA molecules [54].

### Modulating host gene expression in the nucleus

The circRNAs located at the transcription site of the host gene can function by regulating the expression of their host genes. Zhang et al. [41] have found that circRNAs derived from the introns of *ankrd52*, *MCM5*, and *SIRT7* genes are distributed in large amounts in the nucleus. Knockdown of circ-ankrd52 leads to a significant decrease in the expression of *ankrd52* mRNA, indicating that circ-ankrd52 has a regulatory effect on the expression of its host genes. circRNAs also regulate the expression of parental genes in the nucleus by interacting with proteins. For example, circEIF3J and circPAIP2, as nuclear EIciRNAs, can recruit U1 snRNP and Pol II complex onto the promoters of their parent genes to boost the expression of their encoding genes (Fig. 2B) [40].

### Functions of translated circRNAs

Although the coding ability of circRNAs was previously ignored, the translation of endogenous circRNAs into proteins or polypeptides may be a common phenomenon [55]. Some circRNAs bind to polysomes and are actively translated into new short peptides or proteins in a cap-independent manner (Fig. 2C) [56, 57]. This type of circRNAs has an open reading frame (ORF), which increases their translation abilities through the internal ribosome entry site (IRES) or m6A [57–59].

Yang et al. [58]. have found that circ-FBXW7 is driven by IRES and encodes a new functional protein, FBXW7-185aa. FBXW7-185aa is highly expressed in the brains of healthy people. The upregulation of FBXW7-185aa in glioblastoma cancer cells inhibits both the proliferation of tumor cells and the progression of the cell cycle. Knockdown of circ-FBXW7 promotes the malignant phenotype of tumor cells. By analyzing the expression profile of circRNAs of the differentiated mouse and human myoblasts, Legnini et al. [60]. have revealed that circZNF609 is related to heavy multimers and can translate into proteins in a splicing-dependent and cap-independent manner. This is a typical example of circRNA-encoded proteins in eukaryotes. In addition, circRNAs also have coding functions in human cancer diseases [61–63].

### Other functions

In addition to the above-mentioned effects, circRNAs can also regulate the expression of the target genes by interacting with RNA-binding proteins (RBP) (Fig. 2D). Yang et al. [64]. have shown that circ-HuR is a potential target for the treatment of gastric cancer. As a tumor suppressor, circ-HuR interacts with CCHC-type zinc finger nucleic acid-binding protein (CNBP) and therefore inhibits its binding to the human antigen R (HuR) promoter, leading to the downregulation of HuR. Another mechanism of action of circRNAs is to generate cDNA by reverse transcription. The generated cDNA enters the nucleus to destroy the integrity of genomic DNA, thereby exerting pseudogene effects [65]. circRNAs can also be used as potential biomarkers for some diseases [66–68]. For example, hsa_circ_0066755 is significantly increased in the plasma and tissues of patients with nasopharyngeal carcinoma [69] and can be used as an effective diagnostic marker for nasopharyngeal carcinoma.

## circRNAs related to adipocyte metabolism

### Abnormal adipocyte metabolism

Adipocyte metabolism refers to the process of digestion, absorption, synthesis, and decomposition of body adipose tissue with the help of various related enzymes. Abnormal adipocyte metabolism always causes other diseases, such as hyperlipidemia, metabolic syndrome, nonalcoholic fatty liver disease, and atherosclerosis [70–72]. Adipocyte metabolism is a complex physiological process involving a series of genes and regulatory factors. Maintaining the homeostasis of adipocyte metabolism is of great significance for preventing or treating the occurrence of adipogenesis-related disorders. circRNAs exhibit specific expression patterns in different cell types, tissues, and developmental stages, and can affect metabolic processes in vivo [15]. An increasing number of studies have emphasized the roles of circRNAs in adipocyte metabolism and related diseases (Table 1).

**Table 1:** Functional circRNAs involved in adipocyte metabolism.

CircRNA	Mechanism/Partner	Role in fat metabolism	Species	Reference
hsa_circH19	PTBP1	Promotes hADCSs adipogenic differentiation	Human	[16]
circSAMD4A	miR-138-5p/ EZH2	Promotes preadipocyte differentiation	Human	[74]
circARF3	miR-103/ TRAF3	Alleviates mitophagy-mediated inflammation	Mouse	[75]
ciRS-133	miR-133/ PRDM16 pathway	Promotes white adipose browning	Human	[76]
circ_0075932	PUM2	Promoting effect on inflammation and apoptosis in dermal keratinocytes	Human	[77]
circRNA-vgll3	miR-326-5p	Promotes osteogenic differentiation of adipose-derived mesenchymal stem cells	Human	[79]
circRNA CDR1as	miR-7-5p/ WNT5B	Promotes adipogenic and suppresses osteogenic differentiation of BMSCs	Human	[80]
circRNA_013422/ circRNA_22566	miR-338-3p	Regulates osteogenic differentiation of bone marrow stromal stem cells	Mouse	[83, 84]
CircRNA-23525	miR-30a-3p	Regulates osteogenic differentiation of adipose-derived mesenchymal stem cells	Mouse	[85]
circFUT10	let-7c/let-e	Promotes adipocyte proliferation and inhibits adipocyte differentiation	Bovine	[86]
circFLT1	miR-93	Facilitates adipocyte differentiation and suppress proliferation	Bovine	[88]
circRNA_26852	miR-874、miR-486	circRNAs might regulate adipogenic differentiation and lipid metabolism	Pig	[89]
circ-PLXNA1	miR-214/ CTNNB1	Inhibition of circ-PLXNA1 limited the differentiation of duck adipocyte	Duck	[92]
circRNA_0000660	miR-693/ Igfbp1	Reduces liver lipid accumulation	Mouse	[100]
circHIPK3	miR-192-5p	Enhances fat deposition and triglyceride content in HepG2 cells	Human	[101]
circRNA_0046366	miR-34a/ PPARα	Hinders lipid metabolism and cause liver steatosis	Human	[104]
circRNA_0046367	miR-34a/ PPAR*α*	Alleviates hepatic steatosis	Human	[105]
circRNA_021412	miR-1972/ LPIN1	Contributes to the hepatic steatosis via disrupting the balance of lipogenesis and catalytic separation	Human	[106]
circSCD1	JAK2/ STAT5	Promotes the occurrence of fatty liver disease	Mouse	[108]

### circRNAs regulate adipocyte metabolism in adipose tissue

**Fig. 3:**
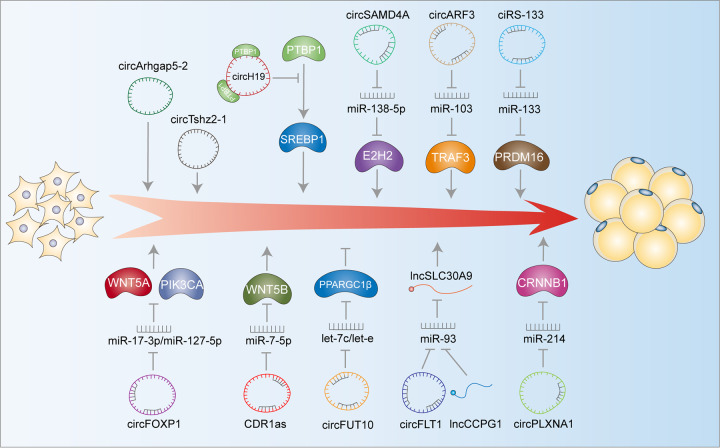
Schematic representation of circRNAs regulating adipogenic differentiation. The arrow represents promotion and the T represents inhibition. circTshz2-1, circArhgap5-2, circH19, circSAMD4A, circARF3, ciRS-133, circ_0075932, circFOXP1, CDR1as, circFLT1, and circ-PLXNA1 promote the differentiation of adipose cells, while circFUT10 inhibits the differentiation of adipose cells.

circRNAs have been found to affect the adipose function of many species, including humans, mice, pigs, and cattle (Fig. 3). Zhu et al. [16] have demonstrated that hsa_circH19 is highly expressed in the blood of patients with metabolic syndrome. Knockdown of the hsa_circH19 gene promotes the differentiation of adipose stem cells by targeting PTBP1. Arcinas et al. [73] have sequenced human visceral and subcutaneous adipose tissue and determined that circTshz2-1 and circArhgap5-2 are indispensable regulators of adipogenesis in vitro. These findings indicate that the expression of circRNAs is essential for maintaining adipocyte metabolism. In human obesity diseases, circSAMD4A acts as a miRNA sponge of miR-138-5p and regulates the expression of EZH2 to control adipogenesis in obese individuals [74]. As a miR-103 sponge, circARF3 inhibits the activity of miR-103 and increases the expression of TRAF3, a downstream target of miR-103. This reduces inflammation in mouse adipose tissue induced by a high-fat diet [75]. Exosomes from gastric cancer cells can deliver ciRS-133 to preadipocytes, and then ciRS-133 promotes the differentiation of preadipocytes into brown adipocytes by activating PRDM16 and inhibiting miR-133, which aggravates cancer-related tumor cachexia. Therapy targeting ciRS-133 may alleviate metabolic disorders in patients with tumor cachexia [76].

circRNAs not only can act as miRNA sponges in adipose tissue but also directly bind to RNA-binding proteins to participate in expression. The expression of circ_0075932 is prominent in human normal adipose tissue. Studies have shown that exosomes released from adipose cells overexpressing circ_0075932 can significantly promote cell inflammation and apoptosis. circ_0075932 promotes cell inflammation and apoptosis by directly binding to the RNA-binding protein PUM2 and promoting PUM2-mediated activation of the AuroraA/NF-kB pathway [77].

Mesenchymal stem cells (MSCs) are highly plastic stem cells, including adipose-derived mesenchymal stem cells (ADSCs) and bone marrow stem cells (BMSCs). They have the ability to self-renew and differentiate into bone cells, chondrocytes, and adipose cells [78]. Recent studies have shown that a significant number of circRNAs are involved in the adipogenesis of MSCs [79–81]. For example, circFOXP1 indirectly regulates EGFR and non-canonical Wnt signaling pathways by sponging miR-17-3p/miR-127-5p to maintain the stemness of MSCs [82]. Knockdown of circRNA CDR1as leads to increased osteogenic differentiation and decreased adipogenic differentiation of BMSCs, while overexpression of CDR1as causes the opposite effect. Mechanismly, CDR1as promotes adipocyte differentiation by competitively binding miR-7-5p with WNT5B [80]. circRNA-vgll3 can directly sequester miR-326-5p in the cytoplasm and inhibits the activity of miR-326-5p, thereby promoting the osteogenic differentiation of ADSCs [79]. miR-338-3p was found to be related to the upregulation of circRNA_013422 and circRNA_22566 during the osteogenic differentiation of mouse adipose-derived stromal cells [83]. miR-338-3p regulates the osteogenic differentiation of mouse bone marrow stromal stem cells by targeting Runx2 and Fgfr2 [84]. circRNA_23525 can regulate the expression of Runx2 by targeting miR-30a-3p and is considered to be an active regulator of the osteogenic differentiation of ADSCs [85].

In animals, Jiang et al. [86] have investigated the expression profiles of circRNAs during the development of bovine adipose tissue, and revealed that overexpression of circFUT10 significantly inhibits PPARγ and C/EBPa expression. circFUT10 functions as a ceRNA for miRNA let-7c/let-e to regulate the differentiation of bovine adipocytes by targeting PPARGC1B. Zhang et al. [87]. have identified that six circRNAs play potential functions during yak adipocyte differentiation. Kang et al. [88] have discovered that circFLT1 and lncCCPG1 are differentially expressed in bovine adipocytes. Overexpression of circFLT1 and lncCCPG1 together promotes adipocyte differentiation and inhibits adipocyte proliferation. By analyzing the expression of circRNAs in the subcutaneous adipose tissue of large white pigs and Laiwu pigs, Li et al. speculated that circRNAs may regulate adipose differentiation and lipid metabolism in pigs [89]. circRNAs are very abundant in pig muscle, adipose tissue, and liver, and are expressed dynamically in a spatiotemporal manner [90]. circLCLAT1, circFNDC3AL, circCLEC19A, and circARMH1 regulate miRNAs through the PPAR pathway to influence the differentiation of chicken adipose cells and tissue-specific adipose deposition [91]. circ-PLXNA1 is mainly expressed in duck adipose tissue and the liver. Inhibition of circ-PLXNA1 limits the differentiation of duck adipose cells [92].

### circRNAs and liver lipid metabolism

The liver plays an important role in the metabolic processes of lipid digestion, absorption, decomposition, synthesis, and transportation. Obesity, excessive drinking, and diabetes may cause lipid metabolism disorders in the liver. Excessive lipid accumulation in the liver generates a large amount of reactive oxygen species in the cells, which causes endoplasmic reticulum stress and mitochondrial dysfunction in hepatocytes and finally induces the occurrence of nonalcoholic fatty liver disease (NAFLD) [93–96]. At present, many circRNAs have been identified to participate in the regulation of lipid metabolism and influence the development of lipid disorders [97, 98].

The consumption of a high-fat diet by the mother alters the expression of miRNAs in the liver of the offspring and impair their metabolic health [99]. Existing researches have discovered another new mechanism of the effect of maternal obesity on liver lipid metabolism in offspring. By performing liver RNA sequencing on the offspring of high-fat diet-fed C57BL/6J mice, Chen et al. [100]. have identified 231 differentially expressed circRNAs, with 121 upregulated and 110 downregulated. The expression of circRNA_0000660 is significantly correlated with the expression of *Igfbp1*, and knockdown of circRNA_0000660 reduces liver lipid accumulation. Maternal obesity certainly causes the offspring to suffer from metabolic disorders and impairs health through altered circRNAs expression levels in the liver. circHIPK3 can regulate the expression of lipid metabolism in hepatocytes. circHIPK3 not only enhances adipose deposition and triglyceride content in HepG2 cells but also causes diabetes-related metabolic disorders, such as hyperglycemia and insulin resistance, by reducing miR-192-5p and upregulating the downstream transcription factor FOXO1 [101].

**Fig. 4:**
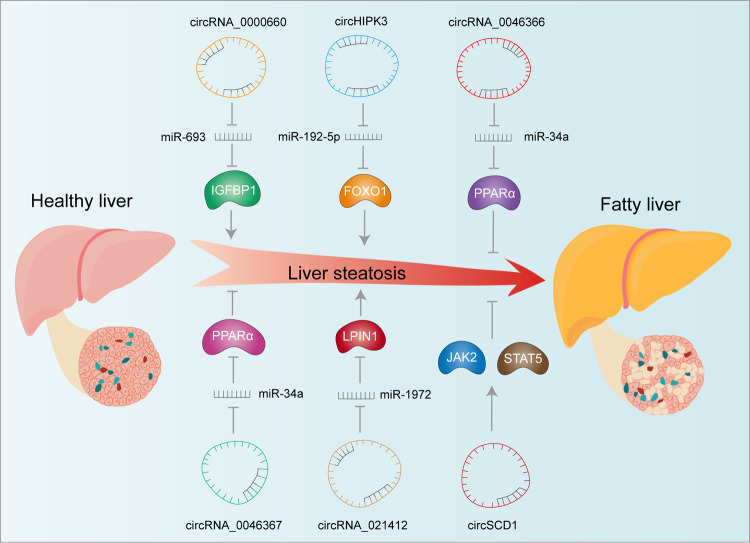
Schematic representation of circRNAs regulating liver lipid metabolism. The arrow represents promotion and the T represents inhibition, circRNA_0000660, circHIPK3, and circRNA_021412 promote lipid deposition by functioning as an ceRNA, whereas circRNA_0046366, circRNA_0046367, and circSCD1 inhibit lipid deposition in hepatocytes.

An increasing number of scholars have revealed the important regulatory roles of circRNAs in hepatic steatosis [102, 103] (Fig. 4). In rodents, the binding of miR-34a and PPARα hinders lipid metabolism and promotes hepatic steatosis, but circRNA_0046366 and circRNA_0046367 can compete with PPARα to bind miR-34a. circRNA_0046366 and circRNA_0046367 are endogenous regulators of miR-34a, and they reduce hepatic steatosis by blocking the interaction of miR-34a and miRNA response element (MRE) located in PPARα mRNA. Abnormal circRNA_0046366, circRNA_0046367/miR-34a/PPARα signal transduction may be a new potential target to treat hepatic steatosis [104, 105]. In high-fat diet-induced hepatic steatosis, circRNA_021412 is significantly downregulated [106]. The decreased expression of circRNA_021412 weakens its competitive inhibition of miR-1972, resulting in the repression of Lpin1, a miR-1972 targeted gene. Decreased Lpin1 expression levels then induce down-regulation of long-chain acyl CoA synthase (ACSL), which ultimately leads to hepatic steatosis.

NAFLD refers to excessive fat deposition in hepatocytes that is not related to heavy alcohol use. A large number of NAFLD patients may further develop nonalcoholic steatohepatitis (NASH) [107]. Abnormal expression of circSCD1 affects lipidation of hepatocytes and promotes fatty liver disease through the JAK2/STAT5 pathway [98]. Jin et al. [97]. have constructed four circRNA–miRNA–mRNA pathways in the NASH mouse model and found that the circRNA sexpression profile can be used the diagnosis of NASH.

## Concluding remarks

With the development of science, technology, and biotechnology, methods to identify circRNAs have also increased rapidly [108, 109]. However, there are still some challenges to studying the expression circRNAs and their related networks in the disease processes [110, 111]. Although there are several independent pieces of evidence that support the miRNAs sponge function of circRNAs, but how to coordinate between the genes, miRNAs and circRNAs requires more comprehensive studies.

circRNAs regulate gene expressions through different targets in different types of diseases and even in different disease stages [112]. Previous studies have provided important evidences for circRNAs as important regulators of adipocyte metabolism. However, in specific diseases caused by abnormal adipocyte metabolism, the expression and regulation of circRNAs needs to be further studied. In addition, the differential expression at the RNA levels does not necessarily indicate a significant difference of related proteins. Therefore, it is necessary to correlate circRNAs and protein analysis to comprehensively study the disease-resistance mechanism of circRNAs. In the future, circRNAs may also be used as biomarkers for metabolic syndrome to predict the occurrence of certain metabolic diseases. However, due to the complexity of metabolic syndrome, the discovery of biomarkers still face multiple challenges [113]. Although some important research results have been obtained in mice, rats, and other biological models [114, 115], its application in human diseases still needs further exploration.

## Data Availability

All data generated or analyzed during this study are included in this published article.
